# Perceptions of Older People with Cognitive Impairment, Caregivers, and Professionals about ehcoBUTLER (Tablet Health Care Platform): A Qualitative Focus Group Study

**DOI:** 10.3390/ijerph19116761

**Published:** 2022-06-01

**Authors:** Leslie María Contreras-Somoza, José Miguel Toribio-Guzmán, María Cruz Sánchez-Gómez, Eider Irazoki, María Victoria Martín-Cilleros, Sonia Verdugo-Castro, Esther Parra-Vidales, María Victoria Perea-Bartolomé, Manuel Ángel Franco-Martín

**Affiliations:** 1 Faculty of Psychology, Campus Ciudad Jardín, University of Salamanca, Avenida de la Merced, 109, 37005 Salamanca, Spain; eider.irazoki@usal.es (E.I.); vperea@usal.es (M.V.P.-B.); mfm@intras.es (M.Á.F.-M.); 2 Department of Research and Development, Iberian Institute of Research in Psycho-Sciences, INTRAS Foundation, Carretera de la Hiniesta 137, 49024 Zamora, Spain; jmtg@intras.es (J.M.T.-G.); epv@intras.es (E.P.-V.); 3 Department of Didactic, Organization and Research Methods, University of Salamanca, Paseo de Canalejas, 169, 37008 Salamanca, Spain; mcsago@usal.es (M.C.S.-G.); viquimc@usal.es (M.V.M.-C.); soniavercas@usal.es (S.V.-C.); 4 Department of Psychiatry and Mental Health, Río Hortega University Hospital, Calle Dulzaina, 2, 47012 Valladolid, Spain; 5 Department of Psychiatry, Zamora Provincial Hospital, Calle Hernán Cortés, 40, 49071 Zamora, Spain

**Keywords:** MCI, dementia, health care, psychosocial stimulation, tablet platform, apps, focus groups, active aging

## Abstract

EhcoBUTLER is a tablet platform mainly aimed at the elderly with mild cognitive impairment (MCI) to promote their well-being and health. The main objective of this study was to explore the perceptions and feedback level of the ehcoBUTLER potential users and stakeholders to improve its development. Through this exploration, the secondary objective was to contribute to the development of software/apps that promote their integral health. Focus groups were conducted (13 elderly with MCI, 13 with dementia, 12 caregivers, 11 professionals). The content and feedback level were analyzed. Participants liked the appearance of ehcoBUTLER, would like to use it, and were mainly interested in the emotional, healthy lifestyle, cognitive, and ergonomic tools. It is necessary to have prior training, more intuitive/customizable apps, low-price/free, offline/USB content, and add other activities/features. EhcoBUTLER is well-oriented to meet the needs and preferences of potential users. However, improvements in its usability, accessibility, and sustainability are needed. The participants’ perspectives provided a comprehensive view to improve ehcoBUTLER, so that in the future, it can benefit the elderly to be active agents in their health; support caregivers in their role and to have a respite; and professionals to have a multi-intervention platform. The present findings can contribute to the development of tablet software/apps that promote the integral well-being of this population.

## 1. Introduction

### 1.1. Background

Older adults often suffer from social isolation, loneliness [1], and cognitive and physical decline [2], which impacts their emotional state [3]. These difficulties may be worsened if cognitive decline progresses to mild cognitive impairment (MCI) and then to dementia [4]. The increase in the life expectancy of the older population represents a challenge to improve their quality of life, while reducing the costs of providing them with care and health services [5]. In this way, technological innovation can be a help and support to promote their well-being and solve this challenge [5].

New technologies have generated a revolution in health and social care [6]. Technologies have facilitated data entry, processing, and storage [7] and have also enabled the automation of activities and processes, leading to greater efficiency and cost reductions [8]. In addition, they have improved flexibility and access to services for people who are homebound, or who have limited transportation [9]. Furthermore, technologies have improved the quality of health care by enabling greater communication between the professionals and patients and dynamic services that increase the patients’ involvement in their health [8]. 

However, the use of new technologies by the elderly also involves challenges, especially if they have cognitive impairment [10]. They face barriers related to the decline in their functions and lack of computer skills because they were born before the technological revolution [11,12]. In addition, they often face problems related to inadequate design, in which their characteristics and needs are not taken into account, so they feel discouraged in using them [10,13]. 

Therefore, technologies that are appropriately adapted to an elderly population with cognitive impairment and that promote their well-being stand out. For example, CIRCA (Computer Interactive Reminiscence and Conversation Aid) is an application (app) for people with dementia (PWD) to interact with photos, video, and music on a touch screen, thus promoting their communication with their caregivers [14] and also improving their quality of life [15]. Another example of technology supporting the care of individuals with MCI or dementia is a virtual reality program with real and familiar environments, which has been shown to improve their mental well-being [16]. 

Regarding the cognitive area, there are various cognitive intervention software for elderly people with MCI or PWD; such a variety allows for the professionals and end users to select the one that best suits their objectives and cognitive needs [17]. In addition, there are games for individuals with MCI or dementia to train cognitive functions such as Kitchen and Cooking, which was considered as interesting and motivating [18]. Furthermore, games can be used for leisure, fun, and recreational purposes [19]. 

On the other hand, the ReaCT (Rehabilitation in Alzheimer’s disease through Cognitive Support Technology) app supports the self-management of PWD through a calendar as the main tool. Functions can be adjusted to suit the needs and preferences of each person, and caregivers also have access to it [20]. Another example is the Digital Memory Notebook (DMN), which is a tablet app to support the independence for individuals with MCI. Most participants perceived that the DMN improved their satisfaction with life and daily activities [21]. 

It has been shown that older adults with MCI and people with early dementia are interested in using technologies that promote their cognitive functions, lifestyle, and overall health [22]. Therefore, a technology that has the tools to address the various aspects above-mentioned could represent several benefits to this population in a single system. 

### 1.2. EhcoBUTLER

EhcoBUTLER is an Information and Communication Technology that has different modules: healthy lifestyle, leisure and free time, emotional, social, and cognitive care. EhcoBUTLER is funded by the European Union (H2020; ID: 643566) and is under development. Its aims to promote the health, well-being, and independence of older people, especially those with MCI at the social level [23]. EhcoBUTLER also has an ergonomic design inherited from BUTLER, which has demonstrated an adequate level of usability in the older population [12]. As part of its ergonomic design, and to promote its portability, the system was especially adapted for use on a tablet. In addition, touch screen tablets are a relatively inexpensive for people with cognitive impairment to engage in meaningful activities [24].

Another important element of ehcoBUTLER is its linear navigation system, which allows users to use the system step-by-step [25]. Users select the apps they wish to use from the main menu, and a human-looking avatar explains in audio and text what they can do next. In addition, the buttons have different colors according to their actions: green is for continuing a task; red is for deleting data or undoing a step; and orange implies secondary actions [10]. 

Family members and informal caregivers of the elderly can access ehcoBUTLER to interact with them and find out what they are doing. In addition, the professionals manage and customize the different modules of the platform and can also use it to communicate with other professionals and relatives of the elderly person.

### 1.3. Human Centered-Design 

Most systems aimed at the elderly population consist of a single app to reduce their mental load; this does not mean that a system with several apps is impossible, but that it requires additional efforts in its design and evaluation [12]. Indeed, elderly people believe that despite the potential benefits of technology, if adequate measures are not taken to help them to use it, the digital divide will expand [26]. It is not only necessary to develop tools that are easy to use, but also to provide a pleasant user experience [27], and a human-centered design would allow for the development of products and services with such features [28]. 

One of the most widely used tools in human-centered design are focus groups [29], because they allow for a better understanding of the needs and particularities of the users, according to their opinions and attitudes [30,31]. Some studies have conducted focus groups of PWD to develop technologies. For example, key aspects in the design of a virtual environment were identified through the opinions and thoughts gathered from focus groups such as easy access, realistic and age-appropriate content, and tasks with specific objectives [32]. In other research, focus groups of PWD provided insights into the challenges they faced in terms of emotional stability and their requirements to develop a sensor platform to monitor their emotional state at home [33]. 

More studies are needed that include older people and investigate how they feel about new technologies, their reasons for using them, their frequency, and form of use [34]. In addition, in the development of technological products and services, the perspectives of the elderly and stakeholders are essential to break down barriers and benefit everyone [35]. The participation of PWD is also a challenge [33], because their communication skills are typically one of the most affected [36]. Their difficulties increase as their disease progresses, so it is important to focus on mild dementia to generate interventions from an early stage [37] as well as from the MCI because of their risk of conversion to dementia [38].

### 1.4. Objective of This Study

The main objective of this study was to explore the perceptions and feedback level of the ehcoBUTLER potential primary users (older adults with MCI and people with mild dementia) and stakeholders (informal caregivers and health professionals) to improve the development of this platform according to their needs, preferences, and suggestions. Through this exploration, the secondary objective was to contribute to the development of tablet platforms and apps aimed at promoting the integral well-being and health of this population.

## 2. Method

### 2.1. Design

This is a qualitative study with a phenomenological approach, in which focus groups were conducted. This approach and technique are useful for gathering and understanding a wide range of perceptions, ideas, and opinions by providing an interactive environment where members share and compare their views, encouraging even reserved individuals to participate [39].

### 2.2. Participants

A total of 49 people participated in the study. Eight focus groups were conducted: two for older adults with MCI, two for PWD, two for caregivers, and two for professionals. Each group consisted of five to eight members to promote their participation [40]. Table 1 details their sociodemographic characteristics.

Potential participants with MCI, dementia, and their caregivers were contacted by telephone, while the professionals were invited by email. They were asked whether they wanted to participate after explaining the aim of the study, the confidentiality of the data, and the estimated duration of the focus groups. In addition, in case any need arose, the caregivers of PWD gave their consent for their relatives to participate.

#### 2.2.1. MCI Groups

They lived in the community and were recruited from the Memory Workshops of INTRAS Foundation (Zamora, Spain). The inclusion criteria were: 55 years or older, have a diagnosis of MCI (meeting the criteria of Petersen [41]) without any associated psychiatric disorder, be able to read and write, and not have vision/hearing difficulties. In addition, the participants had previous experience with a computer-based cognitive stimulation, training, and rehabilitation program called GRADIOR [42,43] from attending those workshops. Thirteen elderly adults with MCI participated. Most of them were widowed (53.85%) women (84.62%) with a primary education (53.85%) and had an average age of 75.31 (SD = 8.05).

#### 2.2.2. Dementia Groups 

Participants were recruited from the Memory Workshops and the Memory Clinic of the INTRAS Foundation (Zamora, Spain). They lived in the community and their inclusion criteria were to be 55 years or older, be able to write and read, have no hearing/vision problems, and have a diagnosis of mild dementia without any related psychiatric disorder. Their diagnosis was based on meeting the Diagnostic and Statistical Manual for Mental Disorders (DSM-5) criteria for major neurocognitive disorder, the current term for which was previously called dementia [44]. 

Participants also had previous experience with GRADIOR from attending workshops or the clinic. A middle-aged adult with early onset of dementia was included as an exception. Thirteen people participated with a mean age of 76.46 (SD = 10.53). Most were widowed (46.15%) women (69.23%) and had a primary education (53.85%). 

#### 2.2.3. Caregiver Groups 

The inclusion criteria for this group were to be informal caregivers who provided instrumental and accompaniment aids to older adults, and to have previous experience with technological devices. Their recruitment also came from the Memory Clinic of INTRAS Foundation (Zamora, Spain). Twelve caregivers participated, most of whom were married (66.67%) women (66.67%), had an associate degree (41.67%), and an average age of age 54.17 (SD = 14.11). 

#### 2.2.4. Professional Groups 

This group had the inclusion criteria of: being a health professional (psychologists, neuropsychologists, therapists, etc.) with experience attending older adults with cognitive impairment and had worked or presently worked with technological tools as part of their clinical intervention. Their recruitment came from different departments of the INTRAS Foundation. Eleven professionals participated with a mean age of 33.09 (SD = 7.91) and most were women (72.73%). 

### 2.3. Procedure

The Provincial Hospital of Zamora gave its ethical approval to carry out this study (Protocol Code: 387-E.C.). The focus groups were conducted at the INTRAS Foundation’s Memory Clinic (Zamora, Spain) and there were two members of the research team during them: one was the moderator, while the other took manual notes.

The moderator began by explaining the objective of the study, the audio and video recording of the meeting, the data confidentiality, and asked the participants whether they agreed to participate. All of them signed the informed consent form and filled out a sociodemographic data form. The same accessible room was used for all groups. To avoid interruptions and distractions, a signboard indicated that the meeting was being recorded.

The moderator then showed a video demonstration of ehcoBUTLER, explained its purpose and functions, and asked the semi-structured questions from the scripts. There were three scripts: one for older adults with cognitive impairment, one for caregivers, and another one for professionals. 

The script of the elderly and that of their caregivers inquired about their use and preference of technologies and social networks, their general perception of ehcoBUTLER and its main tools, aspects to improve it, how often they would use it, and whether they would be willing to pay for it. The professionals’ script differed in asking them about psychosocial stimulation programs, rather than about technologies and social networks.

The moderator redirected the topic when necessary and encouraged all participants to provide their opinion. The focus groups lasted approximately one hour each and, at the end, participants were given a small present to thank them for their collaboration. The verbal content of the discussions was transcribed anonymously for further analysis. All of the material was carefully stored in a security filing cabinet, where only the team’s researchers had access.

### 2.4. Data Analysis

**Figure 1:**
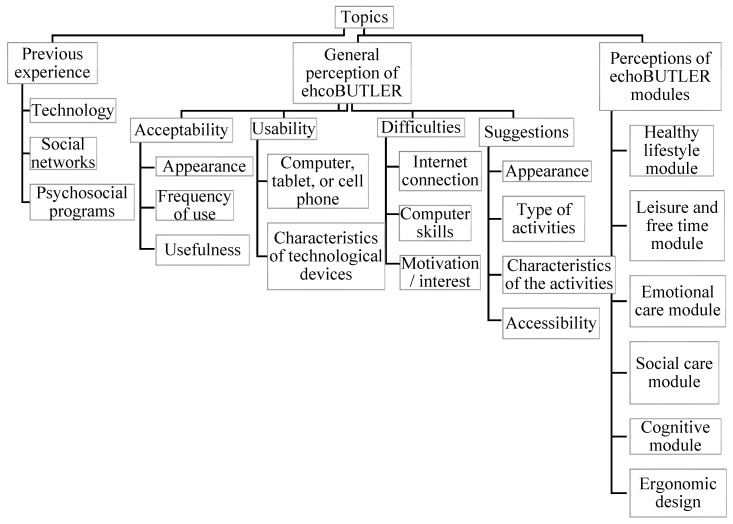
The main categories and subcategories.

**Figure 2:**
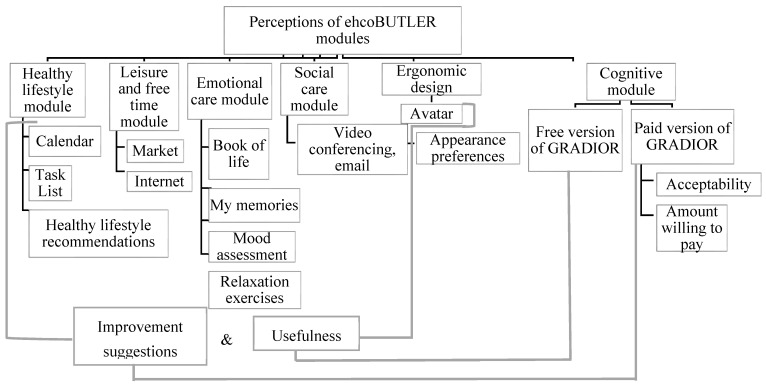
The subcategories of the ehcoBUTLER modules.

First, the sociodemographic data of the participants were analyzed; the SPSS statistical program (version 25) was used to obtain their percentages, means, and standard deviations. Second, the NVivo 12 Plus program was used for the analysis of the focus group data because it makes coding and node management more efficient. Based on the scripts and themes discussed in the focus groups, homogeneous categories and subcategories were created for all groups, in order to have the same coding system and compare the content of the groups in relation to the categories and subcategories. The general categories were: (1) Previous experience with technology, social networks, or psychosocial programs; (2) General perception of ehcoBUTLER; and (3) Perceptions of ehcoBUTLER modules (Figure 1). In addition, due to the extent of the subcategories of the ehcoBUTLER modules, these are detailed in Figure 2.

Third, double entry matrices were created, where the content was crossed between each participant and each of the categories and subcategories to compare the positive or negative perceptions of the groups. 

Fourth, the level of feedback was examined. Considering the degree of clarity, completeness, and elaboration of their answers, a value was assigned for each participant’s response: 1 point for lack/low feedback, 2 points for medium feedback, and 3 points for high feedback. Then, to determine the feedback level of each group, three ranges of scores were established. The minimum and maximum of these scores varied according to the number of members in each group (Table 2). For example, as there were 13 participants in the MCI group, the minimum score had to be 13 and the maximum score 39, so this range was divided into three proportional parts corresponding to the three levels of feedback. 

The procedure described above allowed us to cross the discursive content and the level of feedback in each category and subcategory. Finally, an overall level of feedback per subcategory was obtained by summing the scores with all groups together to determine which were the most commented on topics. These scores were also distributed in the three feedback ranges: low (49–67 points), medium (68–85 points), and high (86–103 points).

## 3. Results

Table 3 summarizes the participants’ positive and negative perceptions and some of their most representative quotes. In addition, Table 4 shows the level of feedback from each group and the most discussed to the least discussed topics.

### 3.1. Previous Experience with Technology, Social Networks, or Psychosocial Programs

The participants presented low to medium feedback on this topic. However, the caregivers provided clear and complete answers (high level of feedback = 29 points) in the technology subcategory, indicating the various devices they had and the uses they gave them, as described below. 

The caregivers had been using cell phones, computers, and/or tablets for personal and/or work purposes (e.g., calls, messages, photographs, Internet) for several years. On the other hand, older adults with MCI and PWD tended to use landlines and/or cell phones to maintain social contact or in the case of emergencies. They also used computers when they attended the Memory Workshops or Memory Clinic. They did not use other technological devices because they did not like them, found them difficult to use, or did not dare. 

The elderly with MCI and PWD did not use any social networks, while the caregivers tended to use Facebook, Instagram, or Twitter. Regarding the psychosocial programs, few professionals reported having experience with these types of programs, finding them easy to use on a tablet and with functions for the elderly person and the caregiver, but with the disadvantage of not being highly personalized.

### 3.2. General Perception of ehcoBUTLER 

#### 3.2.1. Acceptability (Appearance, Frequency of Use, and Usefulness) 

The participants showed medium feedback in this category, showing ideas that were not as developed or that were limited to responding affirmatively or negatively to another participant’s idea. Regarding their perceptions, older adults with MCI, PWD, and the professionals liked the appearance of ehcoBUTLER because of its light colors, the background was not overloaded with elements, and the font type and size. Participants with MCI and PWD stated that they would like to use the platform from every day to a few times a week or in their free time, for an hour or an hour and a half. 

However, the caregivers indicated that their family members would only use it when they attended the center, and not at home, due to their lack of computer skills, cognitive impairment, and that the platform was not very personalized. Professionals also perceived these difficulties. Nevertheless, the caregivers perceived it to be useful in improving the mood of their relatives, even if they did not perform the activities correctly. Older adults with MCI and PWD found it useful to improve their memory, learn new things, relax, or as entertainment. 

#### 3.2.2. Usability (Use of Computer, Tablet or Cell Phone, and Characteristics of Technological Devices) 

Participants presented a low to medium level of feedback on this topic as they responded without explaining the reason for their choice, or their answers could have been further elaborated. Nevertheless, it was possible to identify in their perceptions that people with MCI would prefer to use ehcoBUTLER through a computer. The caregivers preferred for their relatives to use it on a computer or tablet due to the size of their screens. Some professionals supported the tablet for this population because of the intermediate screen size while other professionals supported the cell phone because older adults usually already have one. The PWD also preferred the cell phone for its portability or because it was the cheapest option. However, the participants agreed that the device should have a large touch screen for ease of use compared to a mouse or keyboard.

#### 3.2.3. Difficulties (Internet Connection, Computer Skills and Motivation)

The participants provided low feedback in this category. However, the professionals presented a medium level of feedback (19 points) in the computer skills subcategory, providing some examples of possible difficulties in its use by the population with cognitive impairment, as is shown below. 

The professionals considered it necessary to have computer skills to use the software (chat, password creation, login, enlarge the screen) and hardware (mouse, keyboard), and that it could be difficult for older people; even more so when considering their cognitive impairment, because they could forget important data such as their password. Furthermore, the professionals considered it difficult for elderly people to have the Internet at home, because it is not indispensable for them, and would not be cost-effective for them to pay it just to use ehcoBUTLER. They also pointed out that older adults might be disinterested or discouraged if they perceive its use as an imposition on their family members. 

#### 3.2.4. Suggestions (Appearance, Type of Activities, Characteristics of the Activities and Accessibility)

This category had low feedback from the participants, except in the subcategory of the type of activities where the PWD presented medium feedback (23 points), indicating that it was not necessary to add more activities to ehcoBUTLER. However, the other participants recommended making certain improvements, as described below.

To improve ehcoBUTLER’s appearance, some caregivers suggested the option of customizing it, and some professionals recommended large icons and a small amount of text. Regarding the type of activities, some elderly with MCI wished to add physical exercises and more cognitive exercises. Some caregivers recommended voice command calls and virtual routes for their family members to practice moving from one place to another in their city, even if they could not do it physically. 

Regarding the characteristics of the activities, older adults with MCI wanted to repeat the exercises in which they failed. The caregivers suggested customizing the exercises according to each person’s interests. To improve accessibility, some professionals recommended offline or USB content as well as pre-training for the users. 

### 3.3. Perceptions of the ehcoBUTLER Modules

#### 3.3.1. Healthy Lifestyle Module 

In this category, the participants presented a high level of feedback in the subcategory of usefulness of the healthy lifestyle recommendations (102 points) and the Calendar (92 points), while they gave medium feedback (79 points) on the Task List. Overall, the participants gave low feedback on suggestions for improving these tools. Their perceptions are shown below. 

##### Calendar 

EhcoBUTLER will have a Calendar for entering important dates such as birthdays or doctor’s appointments. Older adults with MCI, PWD, and the professionals perceived it as useful for helping users to remember important dates, given their memory problems. However, some professionals and most caregivers felt that people with cognitive impairment would not be able to use it independently, so they would not enjoy its benefits. 

Several professionals suggested incorporating the prioritization of appointments through eye-catching reminders with sounds and that they should be made a day before the appointment for the elderly and their relatives, even if they were not using the platform. On the other hand, some caregivers recommended that a professional help their relatives to manage this tool.

##### Task List 

In ehcoBUTLER’s Task List, users would be able to record their daily life activities and receive reminders about them. Most of the PWD and some elderly with MCI found it useful for its reminders as they had memory problems. However, one participant with MCI preferred to continue taking notes on paper, and some professionals also supported this, because it would be easier for older adults to take them to where they needed them such as to the supermarket.

Nevertheless, some professionals considered the Task List as being useful because it would allow for elderly people to get into the habit of typing and having everything on the same device. However, some caregivers felt that the cognitive impairment of their relatives would prevent them from using it independently. 

Several professionals suggested that the Task List should be improved by differentiating it from the Calendar or by merging them into one called “reminders”. They also recommended that the platform should send an appointment reminder to the elderly person and the family member at the same time, even if they were outside the platform.

##### Healthy Lifestyle Recommendations

EhcoBUTLER will provide recommendations of healthy habits by proposing activities inside or outside the platform, according to the user’s needs. Most of the older adults with MCI, PWD, and the professionals found the personalized nutritional advice especially helpful. However, most caregivers felt that their relatives would forget the advice or would not apply it in real life. Regarding improvements, several professionals suggested direct access to the advice, motivational notifications of new content, and to keep the previous recommendations available.

#### 3.3.2. Leisure and Free Time Module

The participants provided medium feedback on the usefulness of the Market (84 points) and the Internet (77 points). On the other hand, they had a low level of feedback when suggesting improvements for these tools. Their perceptions are presented below.

##### Market

Through the Market, users are able to incorporate new apps to the system such as games, books, etc. Most older adults with MCI and PWD perceived this as useful for entertainment, learning, and working on their memory, mainly through games or books. However, some participants with MCI preferred activities outside the home such as courses or playing cards.

Most professionals considered that this could be useful if the caregiver selected the activities according to the interests of the older person, in order to facilitate their identification and use. In terms of suggestions for improvement, several professionals suggested providing direct access to the activities previously determined by the caregivers. 

##### Internet

The platform will have an easy and direct access to navigate across different websites. The participants with MCI and PWD wanted to use it to visit websites of interest or to do online paperwork, if it was easy to use and they were taught how to use it. Several professionals also found it useful if access to certain pages was customized to make it easier for the older person to use. Few caregivers felt that this tool could be useful if it had different webpages already set; otherwise, it could be a risky use, for example, pages that involve payments such as hotels.

In terms of suggestions for improvement, few professionals indicated that, in addition to customizing access to certain webpages, the users should have the option of accessing related sites.

#### 3.3.3. Emotional Care Module

The participants provided a high level of feedback on the usefulness of the Book of life, Mood assessment, and Relaxation exercises (88, 103, 99 points respectively), while it was medium on My memories (79 points). There was low feedback in the suggestions for their improvement. These perceptions are shown below.

##### Book of Life

In ehcoBUTLER, users are able to write their autobiography (complementing it with images, audio, video), and share it with their family and friends. Older adults with MCI and PWD felt that this tool would allow them to share with their descendants what they were doing. Some professionals also found it useful for the therapeutic benefits of reminiscence. However, few professionals and caregivers perceived that it could be a burden for the caregiver to digitize the photographs and accompany the family member during the reminiscing process. 

Regarding improvements, some professionals recommended that each person should express their autobiographical memories according to their preferences (writing, speaking, or singing). In addition, one caregiver suggested that the platform narrate them for the elderly. 

##### My Memories

Users will also be able to save photos, music, or videos as memories and will have the option to share them. The participants with MCI, PWD, professionals, and some caregivers considered it useful for evoking meaningful and motivating memories of some moment in the life of the elderly person. However, few caregivers perceived it negatively, because they would not have enough time to help their relatives to elaborate these memories. Regarding possible improvements, few professionals recommended the use of real photographs, and not images downloaded from the Internet. 

##### Mood Assessment

EhcoBUTLER monitors the users’ mood by asking them how they feel when they sign in and sign out. This will allow for the early detection of depressive or anxious symptoms that will alert the professionals. Most of the participants with MCI and PWD perceived it to be useful because it would be a way to take care of them, especially when they were in a low mood when remembering certain events. Several professionals and caregivers also perceived it positively for keeping track of mood, where the professionals added the benefit of being alerted to the situation and adopting content according to it. However, some caregivers and professionals indicated the difficulty that some older people with cognitive impairment might give an automatic response, which did not truly reflect how they felt.

In terms of improvements, several professionals suggested that users should only be asked once a day or every 12 h how they felt to avoid repetitive questions if they accessed the platform several times a day. One caregiver recommended that the question and response options be asked orally, not just in writing. 

##### Relaxation Exercises

These exercises consist of videos about nature with mindfulness techniques. Most of the elderly with MCI, PWD, and half of the caregivers found them useful to help older adults to stop focusing on possible worries or negative thoughts. However, some people with cognitive impairment, a caregiver, and several professionals preferred that the older adults relax by taking walks in nature, which in turn would contribute to their physical health. 

A few participants with MCI suggested incorporating documentaries to relax. Several professionals and a few caregivers recommended that the relaxation exercises be accompanied by scenarios with real images.

#### 3.3.4. Social Care Module

The participants presented a medium level of feedback on the usefulness of videoconferencing and email (83 points), while they presented a low one when suggesting improvements for these tools (56 points). Their perceptions are shown below. 

##### Video Conferencing and Email

Individuals with MCI and PWD as well as the professionals and caregivers felt that videoconferencing would be useful for older adults to make contact and see people who were far away, if it was easy to use. On the other hand, the professionals and caregivers perceived emails negatively, because the population with cognitive impairment would not be able to use it. However, some professionals and a caregiver agreed in recommending that these tools would be improved if the contacts had an icon with their image and direct access. In addition, they suggested that users could send their email message via a voice memo.

#### 3.3.5. Cognitive Module

EhcoBUTLER has two versions of the GRADIOR program: one free and one paid version. Participants gave a high level of feedback (86 points) on the acceptability of the paid version; while they had low feedback on the amount they were willing to pay and on suggestions for improvements. On the usefulness of the free version, they also had low feedback. Their perceptions are described below.

##### Free Version of GRADIOR

This version has cognitive stimulation and training exercises to keep cognitive functions active in general. Older adults with MCI, PWD, and the caregivers considered it useful for working on different cognitive functions, especially memory.

##### Paid Version of GRADIOR

This version contains specialized cognitive training and rehabilitation exercises for impaired cognitive functions, so it may be subject to payment. Some older adults with MCI and PWD had a negative perception about paying for it because they were pensioners, and their financial income was low. However, other participants with MCI were only willing to pay if the amount was small. Several professionals also thought that users might pay if it was a small fee and if they came to a center to encourage adherence by having the support of a therapist. Some caregivers were willing to pay on a month-to-month and wanted the flexibility to decide whether they wanted to continue paying or not, depending on their family member’s level of improvement.

Regarding the amount to be paid, few participants indicated an exact fee: EUR 20 per month estimated by people with MCI; while between EUR 3 to EUR 15 per month, or EUR 300 per year, as estimated by the professionals. As for possible improvements, few professionals suggested that this version should be made available as an add-on within a care package in a nursing home or center to make it more accessible at a flat rate per year.

#### 3.3.6. Ergonomic Design 

The participants had a high level of feedback on the usefulness of the avatar (100 points). On the other hand, they had a medium level on the suggestions for improvement and a low level on its appearance. Their perspectives are shown below.

##### Avatar

This virtual character will assist the users in audio and text at every step of the platform. The participants with MCI, PWD, professionals, and caregivers agreed that the avatar would be useful in facilitating the use of ehcoBUTLER by explaining and reminding the users of the instructions. 

Regarding the characteristics of the avatar as a young man, the PWD indicated that they liked it as it was. Older adults with MCI and caregivers were indifferent to the sex of the avatar; what they considered important was its helping role. However, some professionals and a few caregivers recommended that the avatar have the voice and image of a real person. Other professionals suggested being able to choose whether the avatar would explain the instructions orally, written, or both. 

## 4. Discussion

This was a qualitative study that set up focus groups of older adults with MCI, PWD, professionals, and caregivers to improve the development of ehcoBUTLER, a tablet platform aimed at promoting the health and quality of life in the elderly population. Formally, it should not be considered as just a study of a specific platform, but the evaluation of the main features for the development of tablet-based software for people with MCI or dementia. EhcoBUTLER has different apps to support the independent life and well-being of people with cognitive impairment. There are several systems that are limited to a single app to avoid the mental load on the elderly; however, a system with various apps is also possible, although it requires additional effort to adapt it [12]. The findings of this study can help in the future development of tablet software and apps to promote the integral health of this population.

In general, the participants showed acceptability toward the appearance and usefulness of ehcoBUTLER to promote different areas and were interested in using its apps frequently. The perceived usefulness and interest of the participants of this study aligns with other research in which ehcoBUTLER was considered useful and interesting by potential users and stakeholders [23]. Other investigations have also found that older adults with MCI and PWD are interested in using technologies that promote their health [22], and that aesthetics are important in significantly impacting the perceived suitability of the technology [45].

However, the professionals and caregivers felt that cognitive impairment, lack of computer skills, and lack of motivation of the elderly could make it difficult for them to take advantage of ehcoBUTLER. These results are in line with other studies, which have identified barriers to technological opportunities for the elderly such as their declining functions, lack of technological skills, and may be discouraged from using technology for fear of finding it difficult to use or understand [10,11,13]. In this study, the professionals also pointed out that older adults often do not have access to the Internet. This is supported by a report in which 20–40% of older people in Spain have used the Internet at some time [46]. Therefore, the suggestion of the professionals to provide ehcoBUTLER content offline or via USB is essential to improve its accessibility. In fact, other research supports the alternative, where the elderly can download the apps’ content to access them without an Internet connection [22]. This should be considered in the development of platforms for older people at home.

In this study, the professionals also recommended that potential users receive training to learn how to use ehcoBUTLER. This is important, because participants with cognitive impairment used landlines and cell phones to make calls or in case of emergency and were afraid of using other technologies. Other studies have also identified that receiving training and learning about the benefits of technologies can make the difference in showing interest in them [47] and in striving to master their use, even if they are not perceived as easy at first [48]. Consequently, it is necessary to keep in mind the technological gap in older people and provide them with training to make these technologies familiar to them. This would not only improve the accessibility of the platforms, but also their acceptability and usability. 

To further promote ehcoBUTLER usability, the participants preferred to use it on an inexpensive, portable device with a large touch screen. These results on the characteristics of the technological device are supported by other investigations because touch screens facilitate the interaction of older adults with technological devices [49] and their portability facilitates the application of the intervention [17]. The low cost of the device is also an important consideration, since the incomes of the elderly decrease as they get older [46]. In this regard, touch screen tablets are a relatively low-cost option for the elderly [24]. However, governments should consider providing free apps to promote independent living as a relevant measure to avoid earlier institutionalization. Indeed, another study found that professionals expected the technological intervention to be financed by the National Health System, companies, or centers [23]. 

In the present study, the participants also recommended customizing the platform according to the interests of the elderly as well as adding other activities and features. Another study also highlighted the importance of personalizing the technological tool and taking into account the considerations of the elderly, because it can facilitate a meaningful and positive engagement of the older population with it [50]. Now, we will discuss the apps in ehcoBUTLER where the participants had a high level of feedback and, therefore, a higher level of interest. This will help in the development and improvement of future platforms to contain the apps that most interest this population.

The participants showed a high level of feedback on the usefulness of these tools: Mood Assessment, Healthy Lifestyle Recommendations, Avatar, Relaxation Exercises, Calendar, and Book of Life. The elderly with MCI, PWD, the caregivers, and professionals had a positive perception of their usefulness. At the same time, the caregivers and professionals highlighted that older adults with cognitive impairment would need help to use them and take advantage of their benefits. These difficulties coincide with those they noted in the general perception of ehcoBUTLER, so their suggestions for improving each app as well as customization and training could encourage more independent use. Indeed, research has shown that, in order to break down barriers and benefit everyone, the perceptions of the end users and stakeholders must be considered in the development of technological products and services [35].

In the present study, most of the tools with a high level of feedback belonged to the Emotional Care Module (Mood Assessment, Relaxation Exercises, and Book of Life). Other research has indicated that people with cognitive impairment are more at risk of suffering from depression or anxiety [3,51], so it makes sense that in this study, the participants showed great interest in caring for the emotional and relaxation state of the older adults. In addition, other studies using a reminiscence app [15] and virtual reality [16] found that they improved the well-being and quality of life of the elderly with cognitive impairment. 

Related to the quality of life, the participants of the present study also showed great interest in apps that promoted a healthy lifestyle (Healthy Lifestyle Recommendations and Calendar). There is evidence that cognitive decline influences older adults to feel less able to make decisions about their daily activities [52]. Therefore, it makes sense that in the present study, the participants showed great interest in apps that supported their daily lives, especially in the calendar function and nutritional advice. Other research has supported the recommendations that the elderly maintain a nutritional balance and the use of a calendar to be more oriented in their activities, among others, are useful for them to maintain, as much as possible, one of the most precious capacities: autonomy to take care of themselves and govern their actions [53]. In addition, there is a calendar app [20] and a notebook app [21] that have been reported to contribute to this population’s satisfaction with their lives. 

The participants in this study also had special interest in the avatar, because of its potential usefulness in explaining and remembering what to do on the platform. These results coincide with other research that highlights the avatar’s role as a guide, promoting a positive experience and facilitating its implementation [54]. The fact that ehcoBUTLER will have ergonomic tools that will make its use easier and more enjoyable such as the avatar is a key element for its introduction, especially when the target population is older people with cognitive impairment. This should also be considered in the development of platforms aimed at these target users.

In relation to the cognitive area, the participants showed a high interest discussing the paid version of GRADIOR. The older adults did not want to pay for it or only wanted to pay a small amount because they were pensioners. The caregivers were willing to pay according to the improvement in their family members. These results are consistent with an earlier ehcoBUTLER study [23] in which the end users wished to pay the least amount, while the caregivers wanted to pay depending on its usefulness or preferred it to be financed by other institutions. Once again, the results indicate that the economic issue should be seriously considered in the platforms for this population. 

On the other hand, it is important to note that ehcoBUTLER focused mainly on promoting the social area, but in the present study, the social module did not have a high level of feedback. Nevertheless, the option of sharing the Book of Life (which was highly valued in the Emotional Module) is also part of the Social Module because it involves interaction with other people [23]. The participants’ high interest indicates their preference for this social activity (sharing the Book of Life) rather than videoconferencing or email. This is consistent with other studies in which reminiscence was the most widely used social intervention compared to videoconferencing and others [55]. In addition, email has been found to represent a cognitive load for older subjects due to the various steps they must remember to do tasks such as saving and managing attachments [56]. These aspects should be considered to improve the selection and development of social apps aimed at this population.

In this study, the leisure and free time module apps also did not have a high feedback level. However, in the general perception of ehcoBUTLER, the participants showed interest in its use for entertainment. This discrepancy can be explained by their perceptions that the elderly would need help from their caregivers to configure these tools and preferred them to do activities outside the home. In fact, some participants also supported activities outside the home rather than the Relaxation Exercises in the Emotional Care Module. Activities outside the home are clearly beneficial for the well-being of the elderly, but if they cannot go out due to some limitation, there are activities that they can do at home [57]. In these cases, tablet platforms such as ehcoBUTLER offer them with a good alternative or complement for a more active and healthy life. In this sense, the caregivers play a facilitating role in the use of technology by older adults with cognitive impairment [48]. However, it is important to simplify this task and provide them a respite [50]. This could be achieved with appropriately adapted technologies for the elderly population with MCI or dementia. 

The present study conducted focus groups to gather the points of view of older people with cognitive impairment, caregivers, and professionals to further develop and better adapt the different ehcoBUTLER features. This is line with other studies. It has been shown that end-user and stakeholder perceptions are essential for the ultimate development of the technology to benefit everyone [35]. In addition, focus groups have been proven to be useful to gather and understand their opinions, needs, particularities, and attitudes [30,31]. In fact, in other investigations, the focus groups of PWD yielded key aspects for the design, conceptualization, and refinement of the technologies aimed at this population [32,33]. This technique should be considered in the development of psychosocial stimulation software for the elderly with cognitive impairment.

Finally, it is important to understand the limitations of this study. First, valuable data may have been lost because nonverbal responses were not analyzed. Second, there were no follow-up interviews and some aspects of the focus group dialogue could not be further developed [40], for example, when the participants showed a low or medium level of feedback. Third, the participants already had previous experience with GRADIOR, so their results may be different from those without such experience. However, several studies support the results presented, and knowing a little bit about the uses of technologies was good for their understanding of the objectives of the study. Indeed, having that experience allowed them to have a point of comparison to make suggestions for improvements in ehcoBUTLER or other tablet apps.

Fourth, the participants’ responses could have been influenced by being at the INTRAS Foundation, where they had a relationship with GRADIOR (as users, caregivers of users, or employees of the foundation). However, the platform presented to them in the focus groups was ehcoBUTLER, and its cognitive module was inquired without specifying that it would be GRADIOR. 

Fifth, PWD were included in this study to evaluate ehcoBUTLER, which is especially aimed at older adults with MCI. However, the coincidence of the participants’ perceptions on the positive aspects and features to be improved indicates their acceptability with the required adaptations to be made.

For future work, more studies are needed that focus on the opinions, ideas, and attitudes of older people with cognitive impairment, along with those of the stakeholders, regarding platforms with different apps for health care, wellness, and psychosocial stimulation. In addition, using joint methodologies such as focus groups with interviews, questionnaires, and/or surveys can allow for more knowledge to be acquired for the appropriate development of this kind of tool in a digital era. Future research focused on the above aspects could contribute to providing older adults with MCI/dementia with innovative and adapted platforms that promote their quality of life, in which they could receive care and health services with greater access, efficiency, dynamism, and reduce the costs of such services. 

## 5. Conclusions

The main purpose of this study was to explore the perceptions and level of feedback of the potential primary users (older adults with MCI and PWD) and actors involved in their care (professionals and caregivers) regarding a tablet platform (ehcoBUTLER) to improve its development according to their points of view. In conclusion, the development of ehcoBUTLER is properly oriented, because the participants showed acceptability toward the platform. Its appearance and the tools of the emotional module, healthy lifestyle, cognitive care, and ergonomic design were key aspects in such acceptability. However, usability, sustainability, and accessibility improvements are needed. These improvements could be properly achieved by making ehcoBUTLER apps more intuitive and personalized and offering pre-training to learn how to use them (due to older people’s unfamiliarity with technology) as well as promoting that the platform could be used on large touch screen devices, that had offline/USB content, and was part of a low-cost or free care package. These improvements, being based on the perceptions of the potential users and stakeholders, could encourage users to have a positive experience with ehcoBUTLER and favor its future implementation.

The suggestions for improving ehcoBUTLER were mainly provided by the professionals and caregivers, while the elderly focused on how they felt about the platform. Their different perspectives allows for a more complete vision to advance its development and to better meet their needs and preferences. This could promote that in the future, it could benefit older adults with MCI or mild dementia to be active agents in their own well-being. This could also help caregivers in their role and to have some respite, if the older adults need as little help as possible to use it as well as to provide professionals with a platform for different areas of intervention. Finally, the findings of this study could contribute to the future development and improvement in tablet platforms and apps that promote the integral health of this population.

## Figures and Tables

**Table 1:** The participants’ sociodemographic characteristics.

Participants	Sex	Age	Civil Status	Education Level
P1(MCI)G1	Man	69	Married	Primary school
P2(MCI)G1	Woman	77	Widowed	Secondary school
P3(MCI)G1	Woman	76	Married	Secondary school
P4(MCI)G1	Man	59	Single	Secondary school
P5(MCI)G1	Woman	83	Widowed	Read and write
P1(MCI)G2	Woman	70	Widowed	Primary school
P2(MIC)G2	Woman	87	Widowed	Primary school
P3(MCI)G2	Woman	78	Married	Primary school
P4(MCI)G2	Woman	65	Widowed	Primary school
P5(MCI)G2	Woman	80	Widowed	Read and write
P6(MCI)G2	Woman	82	Divorced	Primary school
P7(MCI)G2	Woman	82	Widowed	Primary school
P8(MCI)G2	Woman	71	Separated	Secondary school
P1(D)G1	Woman	80	Widowed	Read and write
P2(D)G1	Woman	69	Widowed	Primary school
P3(D)G1	Woman	78	Widowed	Read and write
P4(D)G1	Woman	71	Separated	Primary school
P5(D)G1	Man	86	Married	Read and write
P1(D)G2	Man	87	Married	Primary school
P2(D)G2	Man	73	Single	Primary school
P3(D)G2	Woman	77	Widowed	Primary school
P4(D)G2	Woman	50	Separated	University degree
P5(D)G2	Woman	72	Married	Secondary school
P6(D)G2	Woman	77	Widowed	Read and write
P7(D)G2	Woman	81	Married	Primary school
P8(D)G2	Man	93	Widowed	Primary school
P1(C)G1	Woman	51	Single	University degree
P2(C)G1	Woman	36	Married	Associate degree
P3(C)G1	Man	42	Married	Associate degree
P4(C)G1	Woman	37	Married	University degree
P5(C)G1	Woman	49	Divorced	Associate degree
P1(C)G2	Man	73	Married	University degree
P2(C)G2	Woman	78	Widowed	Read and write
P3(C)G2	Man	59	Married	Secondary school
P4(C)G2	Woman	72	Married	Secondary school
P5(C)G2	Woman	50	Married	Associate degree
P6(C)G2	Man	58	Married	Secondary school
P7(C)G2	Woman	45	Single	Associate degree
P1(PR)G1	Woman	27	-	-
P2(PR)G1	Woman	38	-	-
P3(PR)G1	Woman	38	-	-
P4(PR)G1	Woman	29	-	-
P5(PR)G1	Woman	33	-	-
P6(PR)G1	Man	50	-	-
P1(PR)G2	Woman	25	-	-
P2(PR)G2	Woman	35	-	-
P3(PR)G2	Man	39	-	-
P4(PR)G2	Man	26	-	-
P5(PR)G2	Woman	24	-	-

C = caregiver; D = dementia; G = group; MCI = mild cognitive impairment; P = participant; PR = professional.

**Table 2:** The participants’ feedback level scores.

Level of Feedback	Scores			
	MCI	Dementia	Caregivers	Professionals
Low	13–21 points	13–21 points	12–19 points	11–18 points
Medium	22–30 points	22–30 points	20–28 points	19–26 points
High	31–39 points	31–39 points	29–36 points	27–33 points

MCI = mild cognitive impairment.

**Table 3:** An overview of the participants’ perceptions.

Category	Positive Perceptions	Negative Perceptions	Participants’ Quotes
Previous experience with technology, social networks, or psychosocial programs	To communicate, take pictures, use the Internet, and work Easy to use on tablet Functions for the elderly person and the caregiver	Difficulty, fear, or dislike of using it Not widely personalized	P1(D)G1: *“…I don’t dare”* P7(C)G2: *“… for… calls, WhatsApp, taking a picture… checking things on the Internet… for work…”.* P4(PR)G1: *“… Uno Más or Uno Plus… includes a part of care for relatives, medical assistance in taking medication, cognitive rehabilitation and calendar… in tablet format and it was very simple… but you couldn’t individualize it so much…”.*
General ehcoBUTLER acceptability	Simple appearance Appropriate colors and font Daily or frequent use (60–90 min) Emotional, leisure and cognitive benefits	Only use it in a center Lack of computer skills Difficulty due to cognitive impairment Not personalized	P4(MCI)G1: *“I need to be entertained and relaxed with something”.* P5(C)G2: *“I think they would use it here. If they don’t come here, at home they won’t use it”.* P1(PR)G2: *“I liked it, because it has light colors, which do not generate aversion … very simple, that the screen is not full of things …”.*
General ehcoBUTLER usability	Portable Large touch screen Economic	Difficulty using keyboard or mouse	P4(D)G1: *“Tablets are very convenient, because you can take them wherever you want… unless you carry one of those tiny portable computers… if you carry it on your mobile, it is easier…”.* P7(D)G2: *“My opinion is… the one that costs less”.* P3(C)G1: *“Yes, tactile, the bigger the better”.*
General ehcoBUTLER difficulties	-	Internet is not essential for seniors Lack of computer skills Memory problems Disinterested or unmotivated if it is imposed	P4(PR)G1: *“Pay Internet for an application… only for that… if they already have it at home is because they live in a son’s house or because… the council gives access… free…”.* P3(PR)G2: *“… they should know what a chat room is, they should be fluent in using the keyboard, in using the mouse…”.* P1(PR)G2: *“… If they see it as something imposed… they don’t want to”.*
General ehcoBUTLER suggestions	Personalize it Large icons Small amount of text Physical exercises More cognitive exercises Virtual itineraries Voice command calling Repeat exercises Offline/USB content Pre-training for users	-	P2(MCI)G1: *“Yes, a program that makes your head work”.* P3(MCI)G1: *“… exercises with pedals, walking on a treadmill…”.* P7(C)G2: *“… that you can customize it… change the tones, the wallpaper… that it is adaptable for the person… that he/she likes it as much as possible and uses it as much as possible”.* P4(PR)G1: *“That there would be offline content… that they would be provided with a USB with the purchase of the application…”.*
Healthy lifestyle module	Nutritional advice Reminders Appointments noted in one place	Non-independent use Paper notes are easier Similar functions between Calendar and Task List Forgetfulness or disinterest in doing it	P8(MCI)G2: *“It gives ideas of what is good for you and what is bad for you”.* P2(C)G1: *“… my mother doesn’t care if you tell her what to eat, what to walk, what to do… she doesn’t do it…”.* P5(PR)G1: *“… easier for them to take notes on paper…”.*
Leisure and free time module	Entertainment with cognitive benefits Online procedures Visit websites of interest	Non-independent use Risky Internet use	P3(D)G1: *“Good… because it is something you see, and you learn… something always remains in your memory…”.* P4(C)G2: *“…my relative uses it and I am afraid of it because hotels appear. The Internet is very risky, at least for my relative”.* P6(PR)G1: *“… select an application… that would have to be done by an external person, a caregiver, someone who recommends something…”.*
Emotional care module	Reinforcement of identity Relaxation Motivation Emotional state monitoring	Non-independent use Non-genuine answers Virtual environments, not real ones	P2(D)G2: *“I would like to, because then those who come after us will see what we have done…”.* P7(C)G2: *“Very appropriate… something that gives them peace of mind, and that takes them out of a thought that is wearing them out…”.* P4(PR)G2: *“… I think it is much better for them to go to the park for a walk, than to go indoors to see a digital tree when they can go down to the river and see real trees…”.*
Social care module	Social contact, especially with people who live far away	Non-independent use Email difficult to use	P1(MCI)G2: *“Well, I would like it, because there will be someone you have abroad, children, grandchildren…”.* P4(D)G1: *“Good, because if you are also seeing the other caller, then look how nice it is”.* P3(PR)G1: *“I can’t imagine them answering the email… I mean, I don’t see that. I think a videoconference is better”.*
Cognitive module	Exercise cognitive functions, especially memory Personalized Low price	Low economic income	P3(D)G2: *“Yes, anything for the head”.* P1(C)G1: *“It’s fine… as long as it is appropriate to the level of each person, personalized”.* P7(MCI)G2: *“Hey, if it’s not a large amount, because we are pensioners…”.*
Ergonomic design	Explain and remember the instructions	Artificial appearance and voice	P4(D)G2: *“Indicates what to do; good, good, it orients”.* P4(PR)G1: *“… it would be important for the avatar to be as real as possible and less robot-like … for the voice to have the same oscillations as a human person, to have a human aspect …”.*

C = caregiver; D = dementia; G = group; MCI = mild cognitive impairment; P = participant; PR = professional.

**Table 4:** The level of feedback by the subcategories, groups, and totals.

Category	Subcategory		Participants’ Feedback Level				Overall Level of Feedback
			MCI	D	C	PR	
Previous experience	Technology		MF (24)	LF (19)	HF (29)	LF (11)	MF (83)
	Social networks		MF (26)	LF (19)	MF (23)	LF (11)	MF (79)
	Psychosocial programs		LF (13)	LF (13)	LF (12)	LF (14)	LF (52)
General ehcoBUTLER acceptability	Appearance		MF (23)	LF (18)	LF (12)	LF (17)	MF (70)
	Frequency of use		LF (19)	LF (16)	MF (22)	LF (11)	MF (68)
	Usefulness		LF (21)	LF (20)	MF (23)	MF (19)	MF (83)
General ehcoBUTLER usability	Use of computer, tablet, or cell phone		LF (18)	MF (26)	MF (22)	LF (17)	MF (83)
	Characteristics of technological devices		MF (23)	LF (13)	LF (18)	LF (13)	LF (67)
General ehcoBUTLER difficulties	Internet connection		LF (13)	LF (13)	LF (12)	LF (15)	LF (53)
	Computer skills		LF (13)	LF (13)	LF (12)	MF (19)	LF (57)
	Motivation		LF (13)	LF (13)	LF (12)	LF (14)	LF (52)
General ehcoBUTLER suggestions	Appearance		LF (13)	LF (13)	LF (16)	LF (15)	LF (57)
	Type of activities		LF (18)	MF (23)	LF (19)	LF (11)	MD (71)
	Characteristics of the activities		LF (16)	LF (13)	LF (17)	LF (11)	LF (57)
	Accessibility		LF (13)	LF (13)	LF (12)	LF (17)	LF (55)
Healthy lifestyle module	Calendar	Usefulness	MF (27)	LF (21)	MF (26)	MF (22)	HF (96)
		Improvement suggestions	LF (13)	LF (13)	LF (16)	MF (19)	LF (61)
	Task List	Usefulness	LF (15)	MF (25)	LF (15)	MF (24)	MF (79)
		Improvement suggestions	LF (13)	LF (13)	LF (12)	LF (18)	LF (56)
	Healthy lifestyle recommendations	Usefulness	MF (27)	MF (25)	MF (24)	MF (26)	HF (102)
		Improvement suggestions	LF (13)	LF (13)	LF (12)	MF (22)	LF (60)
Leisure and free time module	Market	Usefulness	MF (26)	MF (24)	LF (12)	MF (22)	MF (84)
		Improvement suggestions	LF (13)	LF (13)	LF (12)	MF (20)	LF (58)
	Internet	Usefulness	LF (19)	MF (23)	LF (17)	LF (18)	MF (77)
		Improvement suggestions	LF (13)	LF (13)	LF (12)	LF (14)	LF (52)
Emotional care module	Book of life	Usefulness	LF (21)	MD (28)	LF (18)	MF (21)	HF (88)
		Improvement suggestions	LF (13)	LF (13)	LF (13)	LF (17)	LF (56)
	My memories	Usefulness	MF (22)	LF (19)	MF (22)	LF (16)	MF (79)
		Improvement suggestions	LF (13)	LF (13)	LF (12)	LF (16)	LF (54)
	Mood assessment	Usefulness	MF (29)	MF (22)	MF (27)	MF (25)	HF (103)
		Improvement suggestions	LF (13)	LF (13)	LF (13)	MF (21)	LF (60)
	Relaxation exercises	Usefulness	MF (25)	MF (25)	MF (23)	MF (26)	HF (99)
		Improvement suggestions	LF (15)	LF (13)	LF (14)	MF (21)	LF (63)
Social care module	Video conferencing and email	Usefulness	LF (21)	MF (23)	LF (19)	MF (20)	MF (83)
		Improvement suggestions	LF (13)	LF (13)	LF (14)	LF (16)	LF (56)
Cognitive module	Free version of GRADIOR	Usefulness	LF (16)	MF (23)	LF (15)	LF (11)	LF (65)
	Paid version of GRADIOR	Acceptability	MF (23)	LF (18)	LF (19)	MF (26)	HF (86)
		Amount willing to pay	LF (18)	LF (13)	LF (16)	LF (18)	LF (65)
		Improvement suggestions	LF (13)	LF (13)	LF (12)	LF (17)	LF (55)
Ergonomic design	Avatar	Usefulness	MF (22)	MF (22)	HF (29)	HF (27)	HF (100)
		Appearance preferences	LF (21)	LF (17)	LF (17)	LF (11)	LF (66)
		Improvement suggestions	LF (13)	LF (14)	LF (18)	MF (23)	MF (68)

C = caregiver; D = dementia; HF = high feedback; LF = low feedback; MCI = mild cognitive impairment; MF = medium feedback; PR = professional.

## Data Availability

The data presented in this study are available on reasonable request from the corresponding author.
